# Estimating Water Supply Arsenic Levels in the New England Bladder Cancer Study

**DOI:** 10.1289/ehp.1002345

**Published:** 2011-03-21

**Authors:** John R. Nuckols, Laura E. Beane Freeman, Jay H. Lubin, Matthew S. Airola, Dalsu Baris, Joseph D. Ayotte, Anne Taylor, Chris Paulu, Margaret R. Karagas, Joanne Colt, Mary H. Ward, An-Tsun Huang, William Bress, Sai Cherala, Debra T. Silverman, Kenneth P. Cantor

**Affiliations:** 1 Division of Cancer Epidemiology and Genetics, National Cancer Institute, National Institutes of Health, Department of Health and Human Services, Bethesda, Maryland, USA; 2 Department of Environmental and Radiological Health Sciences, Colorado State University, Fort Collins, Colorado, USA; 3 Westat, Inc., Rockville, Maryland, USA; 4 U.S. Geological Survey NH-VT Water Science Center, Pembroke, New Hampshire, USA; 5 Information Management Services, Inc., Silver Spring, Maryland, USA; 6 Maine Center for Disease Control and Prevention, Augusta, Maine, USA; 7 Dartmouth Medical School, Hanover, New Hampshire, USA; 8 Division of Health Protection, Vermont Department of Health, Burlington, Vermont, USA; 9 New Hampshire Cancer Registry, Concord, New Hampshire, USA; 10 KP Cantor Environmental, LLC, Silver Spring, Maryland, USA

**Keywords:** arsenic, environmental epidemiology, exposure assessment, geographic information systems, water quality modeling, water supply

## Abstract

Background: Ingestion of inorganic arsenic in drinking water is recognized as a cause of bladder cancer when levels are relatively high (≥ 150 µg/L). The epidemiologic evidence is less clear at the low-to-moderate concentrations typically observed in the United States. Accurate retrospective exposure assessment over a long time period is a major challenge in conducting epidemiologic studies of environmental factors and diseases with long latency, such as cancer.

Objective: We estimated arsenic concentrations in the water supplies of 2,611 participants in a population-based case–control study in northern New England.

Methods: Estimates covered the lifetimes of most study participants and were based on a combination of arsenic measurements at the homes of the participants and statistical modeling of arsenic concentrations in the water supply of both past and current homes. We assigned a residential water supply arsenic concentration for 165,138 (95%) of the total 173,361 lifetime exposure years (EYs) and a workplace water supply arsenic level for 85,195 EYs (86% of reported occupational years).

Results: Three methods accounted for 93% of the residential estimates of arsenic concentration: direct measurement of water samples (27%; median, 0.3 µg/L; range, 0.1–11.5), statistical models of water utility measurement data (49%; median, 0.4 µg/L; range, 0.3–3.3), and statistical models of arsenic concentrations in wells using aquifers in New England (17%; median, 1.6 µg/L; range, 0.6–22.4).

Conclusions: We used a different validation procedure for each of the three methods, and found our estimated levels to be comparable with available measured concentrations. This methodology allowed us to calculate potential drinking water exposure over long periods.

Ingesting inorganic arsenic in drinking water is recognized as a cause of bladder cancer [International Agency for Research on Cancer (IARC) 2004; Straif et al., 2009; Subcommittee on Arsenic in Drinking Water 1999; Subcommittee to Update the 1999 Arsenic in Drinking Water Report 2001]. This conclusion is based largely on studies in populations where arsenic levels were relatively high (e.g., ≥ 150 µg/L). The epidemiologic evidence is less clear at the low-to-moderate concentrations typically observed in the United States (Baastrup et al. 2008; Bates et al. 1995, 2004; Cantor and Lubin 2007; Karagas et al. 2004; Mink et al. 2008; Steinmaus et al. 2003).

We conducted a population-based case–control study in northern New England [Maine (ME), New Hampshire (NH), Vermont (VT)] in the United States (Baris et al. 2009). Bladder cancer mortality and incidence rates have long been elevated in this region, and the primary objective of the study was to determine the reasons for this excess. Arsenic, found at moderately elevated levels (generally < 100 µg/L) in water supplies in parts of New England, is among several hypotheses under investigation.

Estimating long-term exposure to arsenic in drinking water is a key study element that requires reconstructing residential water supply sources and arsenic concentrations over the lifetime of a person (Marshall et al. 2007). Although we recognize that other sources of arsenic (e.g., dietary intake) might impact cancer risk, this paper describes the methods used to estimate arsenic concentrations in the water supplies at residences and workplaces of the New England Bladder Cancer Study participants over their lifetime.

## Materials and Methods

Details of the New England Bladder Cancer Study may be found elsewhere (Baris et al. 2009). In brief, we enrolled 1,193 persons who had been diagnosed with bladder cancer from 2001 through 2004 in ME, NH, or VT; 1,418 controls were randomly selected from state-specific Department of Motor Vehicles records (< 65 years of age) or from the Centers for Medicare and Medicaid Services (≥ 65 years of age) and frequency matched to cases by state, sex, and age at diagnosis (within 5 years) (Baris et al. 2009). Written informed consent was obtained from each participant before the study. The study was approved by the review board of each participating institution and in accordance with an assurance filed with and approved by the U.S. Department of Health and Human Services.

We mailed a residence and work history calendar to study participants. We then conducted a home visit and computer-assisted personal interview (Baris et al. 2009), which included recording the exact address of all residences occupied for ≥ 2 years after the age of 10 years. Addresses prior to age 10 were reported by most respondents on the mailed residence history calendar. When exact address information was unavailable, we obtained the most detailed information available (e.g., nearest cross-street or town). We ascertained all jobs held for at least 6 months since the age of 16 years and the town in which each workplace was located. We recorded detailed historical information on residential water supply, including well type and depth for private wells, utility name for public supplies (current home only), and the proportion of water that was drunk at home versus the workplace.

We collected global positioning system (GPS) readings (model 76; Garmin International Inc., Olathe, KS) during each home visit. We used the GPS reading at each current residence (*n* = 2,611), and at past homes (*n* = 448) where wells were sampled to geocode the address in a geographic information system (GIS). We also batch-geocoded the location using ArcGIS (version 9.2; ESRI, Redlands, CA) and Matchmaker SDK Professional software (version 4.3; Tele Atlas, Lebanon, NH). If the GPS and batched geocoded locations were within 500 m of each other and in the same township, we used the GPS location. If not (97 residences), we resolved the location using MapQuest (MapQuest, Inc., Denver, CO) and Google Maps (Google, Inc., Mountain View, CA). For past residences (*n* = 23,277), we used the batch geocoding method followed by manual interactive geocoding techniques to improve the geocoding of addresses that could not be matched exactly. We assigned employment locations (provided only as the town in which the participants had worked) to the centroid of the appropriate census place [U.S. Census Bureau (USCB) 2000]. We created a GIS data layer of all geocoded locations, including attribute data on water supply source (public, private well, and other) that were reported by the participants. Maps of the study area that show current and historical residence locations can be found in Supplemental Material, Figures 1–3 (http://dx.doi.org/ 10.1289/ehp.1002345).

**Figure 1:**
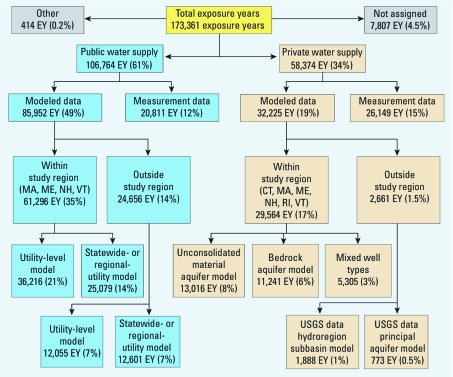
Number and percentage of EYs by each method for assigning residential water supply. “Not assigned” refers to locations outside the United States; “other” refers to EYs for which there was insufficient information to assign.

We estimated the water supply arsenic concentration for each residence for each exposure year (EY) using two basic approaches: direct measurement of arsenic levels in water samples collected at current and selected previous residences, and statistical modeling (Figure 1). We estimated arsenic concentrations at current and past workplaces using similar approaches as those used for previous residences, except we had no direct measurements.

*Direct measurement of arsenic in water supplied to study homes.* During the home visits, we used a commercially washed (mineral-free) polyethylene bottle (Karagas et al. 1998) to collect water samples from 2,599 homes: 1,326 with a public water supply (PWS) and 1,273 with a private supply (99% wells). Samples were not available from 12 current homes. We also sampled water from 448 former homes with private wells, prioritizing bedrock wells in locations with high probability of arsenic > 5 µg/L (Ayotte et al. 2006). All samples were collected from unfiltered home taps after running the water for 1 min. Samples were iced, shipped cold overnight to a repository in Frederick, Maryland, shipped to a laboratory (Dartmouth Trace Elements Analysis Core Laboratory, Dartmouth College, Hanover, NH), acidified with ultrapure nitric acid, and analyzed for dissolved arsenic (as arsenate or arsenite) using inductively coupled plasma mass spectroscopy (model 7500C, Agilent, Santa Clara, CA), within 30 days of receipt. The limit of detection was variable, but < 0.3 μg/L for all samples. Each sample was analyzed twice. We assigned the average arsenic concentration from the two measurements to the period that the participant lived at that home except when the supply to the home had changed. Replicate samples for 253 (~ 10%) of the current home water samples were taken and analyzed concurrent to the primary sample that was used to calculate the coefficient of variation (CV) and to validate direct measurement as an estimation procedure in our study.

*Data sources for statistical modeling of arsenic concentrations in water supplies.* For homes not sampled and for all workplaces, we collected historical arsenic measurement and water source data from public water utilities and historical arsenic measurements for groundwater aquifers.

Arsenic data from public water utilities. For former residences served by a PWS, we assigned the most likely utility. For homes within a USCB place boundary (USCB 2000), we used the boundary as a surrogate for the utility service area (Nuckols et al. 2004a). Homes located outside the place boundary were linked to the utility with the best geographic match. We linked 16,642 residences (90%) reported or assumed to be on a PWS to a specific utility. We requested verification of service from utilities in the primary study states (ME, NH, and VT), and in Massachusetts (MA), a state where 14% of total residential EYs on PWS were assigned in our study. We used data provided by the utilities to evaluate the accuracy of the method. We confirmed service to 81–86% of all residences using PWS; these percentages are consistent with other studies (Luben et al. 2004, 2008; Nuckols et al. 2004a). Verification for 10% of residences served by PWS was not requested, primarily because of incomplete addresses. Addresses not confirmed by utilities were evaluated using ancillary procedures, such as assigning to the most proximate PWS.

For all utilities in MA, ME, NH, and VT, we obtained historical arsenic measurement data and data for current water supply sources (ground, surface, or mixed) from state files. For utilities within this region with the most EYs, we abstracted historical water supply source, water treatment information, and additional measurement data, where available. Outside this region, we abstracted the type of current water supply source (ground and surface) from U.S. Environmental Protection Agency (EPA) data (EPA 2008). PWS-specific arsenic measurements for 1980–2000 were obtained from state agencies in New York, Pennsylvania, Florida, and Connecticut (CT), and abstracted from a U.S. EPA database covering 26 additional states (U.S. EPA 2000). The number of historical measurements available within each of the 30 states covered by one of these data sources is presented by time period in Supplemental Material, Table 1 (http://dx.doi.org/ 10.1289/ehp.1002345). We did not collect PWS measurement data for utilities in 20 other states where estimates of arsenic levels were based on a regional model (4% of total EYs), or for utilities located outside the contiguous United States.

**Table 1:** Assigned residential arsenic concentration (micrograms per liter) by water supply category and EYs.

Percentage of total EYs	Concentration cutpoint by percentile EY in each water supply category										
	Category	25th	50th	75th	90th	95th	Mean
PWS														
Utility model		28.0		0.2		0.4		0.8		2.1		3.8		0.9
State/U.S. EPA region model		21.5		0.4		0.5		0.7		2.2		2.7		0.9
Measurement		12.0		0.2		0.3		0.6		1.1		2.6		0.8
Private well														
Bedrock model*a*		6.5		1.1		2.4		8.8		22.6		30.5		8.2
Unconsolidated model*a*		7.5		0.5		1.2		3.0		5.1		6.7		2.3
Mixed model*a*		3.1		0.6		1.3		4.0		10.8		24.5		4.4
Model outside study region*b*		1.5		0.8		1.4		2.5		4.4		6.0		2.1
Bedrock measurement		11.5		0.1		0.4		2.7		11.8		20.7		5.5
Unconsolidated measurement		2.9		0.1		0.2		0.5		2.3		4.9		1.0
Private supply other measurement		0.7		0.1		0.1		0.2		0.5		1.0		0.4
Unable to assign		4.0		NA		NA		NA		NA		NA		NA
NA, not applicable. **a**Model applies to the six states of New England (NH, ME, VT, MA, CT, RI). **b**Private wells located outside the six-state New England region used for the private well model; estimates made by statistical modeling of arsenic measurement in samples from potable water supply wells (Focazio et al. 1999) and located within the same principal aquifer boundary as past residences in our study.														

Collecting historical arsenic measurement data for aquifers. For homes using private wells in the contiguous United States, we obtained arsenic measurement data from 1971 to 2002 for the predominant potable aquifer in the area of the home from a variety of sources and period ranging from 1971 to 2002 (Ayotte et al. 2006; Focazio et al. 1999; Peters S, personal communication; Smith A, personal communication). For homes in New England [CT, MA, ME, NH, Rhode Island (RI), and VT], the principal aquifer for most current private wells is fractured bedrock (Ayotte et al. 2006). Unconsolidated materials of glacial or glaciofluvial origin (Flanagan et al. 1999), which overlie the fractured bedrock aquifer, provide another source, particularly for pre-1950 wells. For wells < 50 feet deep or construction reported as dug or driven, we designated unconsolidated aquifer as the source type. If > 50 feet in depth and construction was reported as drilled, we assigned fractured bedrock. If we did not know the aquifer type (3% of total EYs), we assigned the average estimated concentration of the two aquifers. For U.S. homes outside of New England, we used the arsenic measurements reported by Focazio et al. (1999) within the principal aquifers [U.S. Geological Survey (USGS) 2008] in which residences were located to estimate arsenic levels in their supply wells.

*Statistical modeling of arsenic concentrations.* We developed models for both public and private water supplies and for unconsolidated and bedrock aquifers. Each model took a similar form:

Ln(As) = **bx** + f, [1]

where Ln(As) is the natural log of the measured concentration of arsenic (micrograms per liter), **x** = (*x*_0_, *x*_1_, …, *x_K_*) and **b** (b_0_, b_1_, …, b*_K_*) are vectors of *K* + 1 regression covariates and their parameters, respectively, and f is the error that we assume is normally distributed with mean zero and variance v^2^; we found this assumption to be appropriate within each type of water source based on our examination of quintile (Q-Q) plots within subgroups that were defined by the type of water source. Because measured data included values below detection limits (BDL), we could not use standard linear least squares regression. Thus, we fitted parametric Tobit regression models that allowed for left-censored data (Lubin et al. 2004). When a measurement was reported as BDL with no reported detection limit, we assumed the following limits of detection in our model: before 1995, 5 μg/L; from 1995 to 2000, 1 μg/L; and from 2001 forward, 0.5 μg/L, based on reported limits from other utilities during these time periods. A detailed description of each prediction model used to assign arsenic to the water supply of residences and workplaces is available in Supplemental Material, Table 2 (http://dx.doi.org/ 10.1289/ehp.1002345).

**Table 2:** Percentage of EYs by arsenic concentration in residential water supply and by attained age of study participant, truncated to reference date.

Age category (years)										
Arsenic concentration (µg/L)	< 10	10–20	> 20–30	> 30–40	> 40–60	> 60–79
< 1		65		63		61		66		68		74
1 to < 3		18		18		18		18		17		12
3 to < 5		5		5		5		6		5		4
5 to < 7		2		2		2		2		2		2
7 to < 10		1		1		2		2		2		1
≥ 10		3		2		2		4		5		6
Not estimated		6		8		11		3		2		1
EYs with arsenic estimated		24,543		26,423		23,499		25,148		45,419		19,947
EYs with arsenic not estimated		1,567		2,298		2,611		778		927		201
Total number of EYs		26,110		28,721		26,110		25,926		46,346		20,148

For a covariate vector, **x**, we computed the predicted value for arsenic concentration as exp(*bx* + 0.5*s*^2^), where **b** and *s*^2^ were estimates of **b** and v^2^, respectively. Depending on the model, **x** included continuous variables or indicator variables for categorical variables. Several models of geographical areas such as states and USGS hydroregions (Watermolen 2005) included only categorical variables. For these models, we omitted the intercept, so that exp(*b_k_* + 0.5 × *s*^2^) was the predicted arsenic concentration for level *k* of the geographical unit.

Former homes on public water supplies inside and outside the four-state region. For homes on PWS in the four-state region (MA, ME, NH, and VT), we used measurements to fit four different models for predicting arsenic concentrations in the water supplies. Each model corresponded to a water source category (surface water, unconsolidated groundwater aquifer, bedrock groundwater aquifer, or mixed surface and groundwater). Outside the four-state region, we fitted geography-based categorical models to PWS data; however, there were sufficient data for only two types of water supply: groundwater and surface water sources (U.S. EPA 2008).

For PWS models with only categorical variables, we could not directly estimate b*_k_* in two circumstances. First, when all measured values, *n*, for utility *k* were BDL, we used a summary estimate of *s*^2^ and selected a value for *b_k_* based on a binomial distribution (nondetected or detected) such that there was a 0.9 probability of selecting a sample of size *n* with zero successes. Second, when there were no data for a utility, we predicted arsenic concentration using a state-specific model of measurement data from utilities with the same type of water source, weighted by service population. When state-specific data were not available, we used a model based on U.S. EPA regional data (U.S. EPA 2009). These approaches assumed that utilities with measurement data were representative of utilities using the same type of water supply source within the entire state or region.

Former homes with private water supplies in the six New England states. For private water supplies within the six New England states (CT, MA, ME, NH, RI, and VT), we developed separate models for bedrock and unconsolidated materials aquifers using continuous and categorical covariates (*x*) derived for the locations of participants’ wells. Based on the general form of Equation 1, we set *x*_0_ = 1, so that b_0_ was the intercept parameter and exp(*b_k_*) represented the estimated proportional change in As per unit change in *x_k_*.

There were 12 variables in the bedrock aquifer model and 13 in the unconsolidated materials aquifer model. Variables included multiple level variables—geologic provinces, lithochemistry (of bedrock units), and bedrock units (surficial geology)—as well as geochemical and geographic factors associated with the occurrence of arsenic in New England aquifers. These covariates are more fully described elsewhere (Ayotte et al. 2006). A complete listing of the variables used in these models is available in Supplemental Material, Table 3 (http://dx.doi.org/ 10.1289/ehp.1002345).

**Table 3:** Percent agreement between dichotomous classifications based on predicted and observed arsenic concentrations in 1,449 bedrock residential wells in Maine, New Hampshire, and Vermont.

Exposure classification cutpoint concentration													
≤ 2 or > 2 µg/L				≤ 5 or > 5 µg/L				≤ 10 or > 10 µg/L			
Buffer radius (km)*a*	None	10	7	5	None	10	7	5	None	10	7	5
Overall agreement*b*		54	61	63	42		57	63	66	70		65	74	78	80
Specificity*b*		38	50	57	65		52	61	67	73		66	78	83	86
Sensitivity*b*		91	85	77	65		78	71	66	58		62	50	43	36
**a**Predicted concentration by our model is constrained to within the range in concentration measured in samples from other bedrock wells within each specified buffer distance from wells used for model validation. “None” indicates no constraint was employed. **b**The observed level of arsenic in each well was used as the reference concentration in each comparative analysis. Specificity indicates the percentage of wells with predicted values less than or equal to the cutpoint when the measured value is less than or equal to the cutpoint; sensitivity indicates the percentage of wells with predicted values greater than the cutpoint when the measured value is greater than the cutpoint.															

Former homes on private water supplies outside the six New England states. We constructed categorical models to estimate arsenic concentrations in residential wells located within each USGS hydroregion subbasin (Watermolen 2005) based on arsenic concentrations in drinking water wells (Focazio et al. 1999). If no measurement data were available for a specific subbasin, an aquifer-specific model was constructed using data from subbasins wholly contained within a USGS-designated principal aquifer (USGS 2002, 2008).

Assessment of models. For public water supplies within the study area, we compared predicted with observed arsenic levels in utilities where we had current home measurements. Because these measurements were included as input data for parameter estimation (Equation 1), we were unable to perform a formal statistical validation. Instead, we qualitatively evaluated model performance by comparing predicted with observed arsenic levels overall and by model category (surface water, unconsolidated materials groundwater source, mixed surface and groundwater, and unspecified aquifer groundwater). We restricted our assessment to the 98 utilities with three or more current home measurements.

For former homes in New England with private water supplies, we tested the validity of the model estimates using sequestered data sets of measured arsenic concentration in current and selected past home wells of study participants (1,449 from bedrock aquifer wells, 282 from unconsolidated aquifer wells). We used the observed level of arsenic as the reference and computed overall agreement, sensitivity, and specificity for analytical cutpoints of 2, 5, and 10 µg/L. We also assessed the validity of a modified modeling approach where predicted arsenic concentrations were constrained to be within the range of measured arsenic levels from wells within 5, 7, and 10 km of each validation well and evaluated how each constraint affected these statistics. Finally, we calculated the Kendall rank correlation between predicted and observed concentrations in the entire validation data set.

*Evaluating residential and workplace water supply contributions to arsenic exposure.* We estimated arsenic levels in both the residential and workplace water supplies for the 2,479 respondents and 82,484 (47%) EYs for which these data were both available. We evaluated the relative home and workplace contributions using two approaches. We compared the estimate of arsenic concentration in the home with the mean arsenic concentration for the combined home and workplace estimates, weighting by the proportion of drinking water derived from each location (from the interview). We calculated the Kendall rank correlation between these two estimates. We used SAS software (version 9.2; SAS Institute Inc., Cary, NC) for each of the statistical analyses in this assessment.

## Results

*Estimates of lifetime arsenic concentrations.* We assigned a specific water supply and arsenic concentration to 165,138 (95%) of the 173,361 residential EYs in the study, and to 85,195 (86%) of the 99,049 reported workplace EYs. Remaining EYs primarily had missing information from residential or work histories.

Geocoding residential and workplace location. We geocoded 95% of the residential addresses to a postal service ZIP code boundary (median area 75 km^2^); 69% were geocoded to an exact street address or nearest intersection; 1% were geocoded to a U.S. county or state. We did not geocode 4% of total EYs because the address was located outside of the United States, missing, or not geocodable. We geocoded workplace location for 91,125 EYs (92% of reported occupation years). The remaining 8% of reported occupation years were in a workplace located in foreign country or aboard a ship or had incomplete information.

Arsenic concentration estimates by water supply type and age of participants. We assigned arsenic concentrations for 46,960 EYs (27%) using measurements of water samples from the homes of the participants. The interquartile ranges (IQRs) for the measured values were 0.2–0.6 µg/L for PWS, 0.1–2.7 µg/L for private bedrock wells, 0.1–0.5 µg/L for private unconsolidated wells, and 0.1–0.2 µg/L for other private supplies (Table 1). Assigned concentrations for 85,952 EYs (50%) were from statistical models of utility measurement data, either from the utility linked to the residence location (48,271 EYs, 28%) or from the utilities in the state with the same water supply source (ground or surface) as the residence (37,680 EYs, 22%). The overall IQR of concentrations predicted by PWS utility models was 0.2–0.8 µg/L. Assigned concentrations for 32,225 EYs (17%) were derived from regression models of arsenic concentration in private wells in New England (IQR 0.5–8.8 µg/L). Assigned arsenic levels were higher in bedrock aquifer private wells in New England than in other water source types.

Table 2 shows the percentage of EYs by residential arsenic concentration and by attained age. We assigned arsenic concentrations for ≥ 90% of EYs when participants were ≤ 30 years of age, and for ≥ 97% of EYs when they were > 30 years of age. The percentage of EYs with arsenic concentrations of 5 to < 10 µg/L were similar (3–4%) for all age categories. The proportion of EYs with arsenic estimates of ≥ 7 µg/L increased from ≤ 4% for EYs at < 30 years of age to 7% at 41–80 years of age. The change in the exposure distribution with age corresponded to an increased use of private wells after 1960 and an increased use of bedrock rather than unconsolidated aquifers for private wells in New England. Private wells contributed about 35% of EYs in 1930–1939, declining to 25% in 1950–1959, and increasing to 28% in the 1960s. Private wells accounted for 48% of EYs during the years of our field study (2001–2004).

For the 85,195 workplace EYs for which we assigned an arsenic level, estimates of median workplace concentration were 0.5 µg/L (IQR, 0.3–1.2; 72,003 EYs) for PWS and 2.4 µg/L (IQR, 0.9–9.6; 9,321 EYs) for private wells. For the 82,484 EYs with an assigned arsenic concentration for both residence and workplace, the supply type (PWS vs. private well) was the same for 53,615 (65%). For 22,987 EYs (28%), the PWS utility was the same for both residence and workplace. For 18,095 EYs (22%), participants had a private well at home and a public supply at the workplace. We compared home and workplace arsenic among the 2,479 respondents with estimates of home and workplace arsenic levels over the same years; the median concentration was 0.8 µg/L for the home supply (IQR, 0.4–1.9) and 0.7 µg/L for the workplace (IQR, 0.4–1.6). When we compared estimated arsenic levels in the water supply of these participants (residential levels only vs. a weighted residential–workplace level), the Kendall rank order correlation coefficient was 0.8 (*p* < 0.0001).

*Assessment of estimation methods.* Direct measurement of water samples. The CV was 18.0% in the comparison of arsenic concentration in primary versus replicate water samples collected at 253 participant residences (~ 10%) at the time of the home interview.

Public water supplies within the four-state region (MA, ME, NH, and VT). We included current home measurements in PWS models for 124 utilities, accounting for 16% of total EYs. For the 98 utilities with three or more measurements, 74 (76%) had a predicted concentration within the range of the measured concentrations for the utility. The model for utilities using surface water sources performed best in this regard in 45 of 48 utilities (93%). The 75th percentile of the measured concentrations was < 2.4 µg/L across these 48 utilities. The model for utilities using unconsolidated materials aquifers as water source performed worst, with 50% of the 24 utilities having a predicted concentration within the range of the measured concentrations. The 75th percentile of the measured concentrations for these utilities was < 3.3 µg/L. Across all water source types, when a predicted concentration was outside the range of measured concentrations, the predicted concentration was always greater than the highest measured concentration. However, these predictions were all < 1.2 µg/L. Estimates using statewide models accounted for 14% of total EYs and followed a similar pattern in that the medians of predicted concentrations were usually higher than the respective medians of arsenic measurements reported by utilities within a water source category (data not shown), and in all cases were < 0.7 µg/L (Table 2).

Bedrock wells in the study area. For residential wells using the bedrock aquifer in the study area, overall agreement between exposure classification based on an arsenic concentration cutpoint of 10 µg/L (predicted vs. observed concentrations) was highest (80%) when the predicted concentration by the model was constrained to within the range of concentrations in other bedrock wells within 5 km of the model validation wells (Table 3). For classification based on a cutpoint of 2 µg/L, the highest overall agreement (63%) was observed when the predictions were constrained to concentrations in wells within 7 km. For a cutpoint of 5 µg/L, the highest overall agreement (70%) was achieved when the model predictions were constrained to concentrations measured in wells within 5 km of the validation wells. The maximal specificity of our bedrock well model for all classification cutpoints was attained when predicted concentrations were limited to those measured in other bedrock wells within 5 km of the validation wells, and specificity increased as cutpoint concentration increased. Sensitivity, however, was highest when no such constraints were imposed on predicted arsenic concentrations by our model. The Kendall correlation coefficient was 0.28.

Unconsolidated aquifer wells in the study area. For wells using unconsolidated aquifers in New England, the overall agreement between predicted and observed levels was 56, 86, and 96% for our classification cutpoints of ≤ 2, ≤ 5, and ≤ 10 µg/L, respectively. Specificities were 58, 91, and 99% for the same cutpoints. Because no predicted level exceeded 5 µg/L, we could calculate sensitivity only for the ≤ 2 µg/L cutpoint (40%). The percentage of measured concentrations above each cutpoint in our validation data set was 14, 5, and 2% for 2, 5, and 10 µg/L, respectively. The Kendall correlation coefficient was 0.13 when no geographic constraints similar to those for bedrock wells were imposed on the predicted concentration.

## Discussion

Our estimates of arsenic concentrations for the study subjects encompassed 95% of cumulative lifetime residential histories, which is higher than many other studies of arsenic and cancer risk (Baastrup et al. 2008; Bates et al.1995; Kurttio et al. 1999; Steinmaus et al. 2003). A similar level of estimates was achieved in a study of controls from a bladder cancer study in Michigan (Meliker et al. 2007). However, in that study, past residences outside the study area were not geocoded and were assigned a low concentration (0.3 µg/L) if PWS or private well data were not available for areas where subjects had lived. In our study, differences in arsenic levels between public and domestic wells were not consistent across the United States. We found substantial variation in arsenic concentrations in both PWS and private well estimates for past homes, indicating the assignment of a single concentration across all water supply source types could result in exposure misclassification. Kumar et al. (2010) reported a similar difference between public and private wells within regions of the United States. These findings should be taken into account in exposure assessment.

We estimated arsenic concentrations in residential water supplies for at least 80% of the individual lifetime histories for 92% of our study population. Three sources of data accounted for 93% of the EYs with arsenic estimates: direct measurement at residences of the subjects (27%), statistical modeling of PWS measurement data (49%), and predictive models of arsenic concentrations in private wells in New England (17%). The high degree of residential mobility in the study population and the temporal increase in private well use of the more arsenic-laden bedrock aquifer later in life resulted in average lifetime concentration ranges narrower than are apparent from the distributions shown in Table 1.

A common practice in retrospective studies of drinking water arsenic and bladder cancer is the use of relatively recent measurement data to estimate historic concentrations (Baastrup et al. 2008; Bates et al. 1995, 2004; Meliker et al. 2007; Steinmaus et al. 2003); accuracy of these estimates depends, in part, on the temporal stability of arsenic levels and the accuracy of the measurements. Arsenic levels in New England bedrock wells are relatively stable (Ayotte et al. 2003; Karagas et al. 2001), and we assumed low temporal variation in concentration in most PWS in the region within water source type. We assigned arsenic estimates to EYs where we measured samples from the current homes of the respondents for as long as the participant was living there with the same water source. If PWS was the supply, then we used only the measured value for years when the PWS was using the same water source type as when we collected the water sample.

We compared model-based estimates of arsenic for 98 of the larger utilities in the three-state study region (ME, NH, and VT) with measured arsenic in homes served by the respective public supplies and found them to be congruent for PWSs with surface water sources. However, there was less congruence for PWSs with groundwater sources, especially if the source was unconsolidated materials aquifers. In these instances, measurement data were used in model development, and we lacked an independent database for a formal validation. The median and mean levels of both modeled and measured arsenic in water from PWS were < 1.0 µg/L, and the 90th percentiles were < 2.2 (Table 1). Given these assessment results and the low concentration range among PWS in relation to much higher estimates for private wells (Table 1), differences in actual and predicted PWS arsenic levels should not have meaningful consequences for exposure assessment.

Our model for past arsenic levels in New England private wells on bedrock aquifers (6% of total EYs) evolved from an approach in which we used logistic regression to predict the probability of arsenic > 5 µg/L (Ayotte et al. 2006). The overall agreement between measured and predicted levels for the logistic model was approximately 80%, with 37% sensitivity and 93% specificity. When categorizing exposure > 5 µg/L and ≤ 5 µg/L, overall agreement of our prediction model was 57%, with 78% sensitivity and 52% specificity (Table 3). When we compared predicted classifications with those based on measurements in wells within 5, 7, or 10 km, we improved this overall agreement and specificity to as much as 70% and 73%, respectively, but lowered sensitivity (Table 3). Our model for unconsolidated strata wells exhibited low specificity (58%) for classification using an analytical cutpoint of 2 µg/L, but relatively high specificity (91%) for classification using a cutpoint of 5 µg/L. Of the 1,027 bedrock wells serving current homes, we measured arsenic concentrations > 5 µg/L in 178 wells (17%). Of 206 wells in unconsolidated strata, only 11 (5%) were > 5 µg/L. Such a low prevalence of potential exposure generally suggests use of a model with high specificity to minimize false negatives in epidemiological risk calculations (Nuckols et al. 2004b; Stewart and Correa-Villasenor 1991). Considering the specificity of our models, a cutpoint of 5 µg/L could be useful for exposure classification in an epidemiological risk analysis concerning arsenic levels in water supply in our study area.

Our bedrock and unconsolidated models enable the prediction of a unique concentration for each residential and workplace well, allowing flexibility in epidemiologic analyses beyond that provided by a dichotomous metric. Private wells provided the highest potential for exposure to arsenic in drinking water in our study (90th percentile of the measured arsenic concentration for bedrock wells was 11.8 µg/L) in our study. The bedrock aquifer in New England has been used increasingly since 1960 and was the most common source for current study homes using private wells (approximately 80%). Before the advent of mechanized, rotary drilling in the 1950s, homes with private wells drew primarily from unconsolidated strata with lower arsenic concentrations (90th percentile = 2.3 µg/L).

We found a difference in water supply source (public vs. private) between the residence and workplace for about one-third of the EYs for which we could estimate an arsenic concentration. Although the correlation between residential and workplace estimates was high (0.8, *p* < 0.0001), the absolute differences in workplace arsenic concentration range were considerable. It could be that the large difference in EYs (85% vs. 15% for the 85,195 workplace EYs for which we assigned an arsenic level) between much lower concentration PWS sources (IQR, 0.3–1.2 µg/L) and higher concentration private well sources (IQR, 0.9–9.6 µg/L), respectively, obscured these differences in the statistical analysis. There was also uncertainty in the compared data, because we had direct measurements for some of the residences, but we had none for the workplaces. In addition, the spatial resolutions of the geocoding differed for residences versus workplace, and the overall concentrations are very low across both datasets. These limitations could cause spurious or contradicatory results. Therefore, we suggest further research is warranted to evaluate whether arsenic estimates in residential water supplies are generally adequate for estimating exposure in workplaces, as they appear to be for our study population.

Although great effort was undertaken for this exposure assessment, as with any retrospective assessment, there are inherent limitations that should be acknowledged. For example, we estimated arsenic levels by water source type across a lifetime exposure period for most study participants. Uncertainty of these estimates may be substantial because of imprecision and inadequate spatial resolution of statistical models, limited data from PWS, and other factors, such as the assignment of inaccurate laboratory detection limits for arsenic concentrations used in our models. Our estimation procedures were conducted blindly with regard to disease status, suggesting that exposure misclassification would likely bias estimates of risk toward the null. Another important limitation is the low range in concentrations across the lifetime of most study participants, even though the study was conducted in a region with relatively high levels of arsenic in some groundwater aquifers and where a substantial proportion of the population uses private wells. This can be attributed to the high degree of mobility in the study population, an important consideration in environmental epidemiological study design.

In summary, we have described an extensive approach for estimating arsenic concentrations in the residential and workplace water supplies of 2,611 participants in the New England Bladder Cancer Study. We derived location information for 24,583 reported lifetime residences and 14,587 workplaces. We estimated arsenic concentrations for 95% of the 173,361 EYs of study subjects and for 86% of the 99,128 EYs represented by workplace histories. Capturing this degree of lifetime variation in water supply sources, and thus arsenic concentrations, resulted in lower potential lifetime exposure estimates than perhaps expected considering the high groundwater concentrations and proportion of private well use in the selected study area. The location and water supply source information developed by our study will be valuable in subsequent studies of the association between other drinking water and environmental contaminants, bladder cancer, and possibly other health end points.

## Supplemental Material

(444 KB) PDF

## References

[r1] Ayotte JD, Montgomery DL, Flanagan SM, Robinson KW (2003). Arsenic in groundwater in eastern New England: occurrence, controls, and human health implications.. Environ Sci Technol.

[r2] Ayotte JD, Nolan BT, Nuckols JR, Cantor KP, Robinson GR, Baris D (2006). Modeling the probability of arsenic in groundwater in New England as a tool for exposure assessment.. Environ Sci Technol.

[r3] Baastrup R, Sørensen M, Balstrøm T, Frederiksen K, Larsen CL, Tjønneland A (2008). Arsenic in drinking water and risk for cancer in Denmark.. Environ Health Perspect.

[r4] Baris D, Karagas MR, Verrill C, Johnson A, Andrew AS, Marsit CJ (2009). A case–control study of smoking and bladder cancer risk: emergent patterns over time.. J Natl Cancer Inst.

[r5] Bates MN, Rey OA, Biggs ML, Hopenhayn C, Moore LE, Kalman D (2004). Case–control study of bladder cancer and exposure to arsenic in Argentina.. Am J Epidemiol.

[r6] Bates MN, Smith AH, Cantor KP (1995). Case–control study of bladder cancer and arsenic in drinking water.. Am J Epidemiol.

[r7] Cantor KP, Lubin JH (2007). Arsenic, internal cancers, and issues in inference from studies of low-level exposures in human populations.. Toxicol Appl Pharmacol.

[r8] Flanagan SM, Nielsen MG, Robinson KW, Coles JF (1999). Water Quality Assessment of the New England Coastal Basins in Maine, Massachusetts, New Hampshire, and Rhode Island—Environmental Settings and Implications for Water Quality and Aquatic Biota. Water Resources Investigations Report 98-4249.. http://pubs.usgs.gov/wri/wri984249/.

[r9] Focazio MJ, Welch AH, Watkins SA, Helsel DR, Horn MA (1999). A Retrospective Analysis on the Occurrence of Arsenic in Ground-Water Resources of the United States and Limitations in Drinking-Water-Supply Characterizations. U.S. Geological Survey Water-Resources Investigation Report 99-4279.. http://pubs.usgs.gov/mirror/wri/wri994279/.

[r10] IARC (International Agency for Research on Cancer) (2004). Some drinking-water disinfectants and contaminants, including arsenic.. IARC Monogr Eval Carcinog Risks Hum.

[r11] Karagas MR, Le CX, Morris S, Blum J, Lu X, Spate V (2001). Markers of low-level arsenic exposure for evaluating human cancer risks in a U.S. population.. Int J Occup Med Environ Health.

[r12] Karagas MR, Tosteson TD, Blum J, Morris JS, Baron JA, Klaue B (1998). Design of an epidemiologic study of drinking water arsenic exposure and skin and bladder cancer risk in a U.S. population.. Environ Health Perspect.

[r13] Karagas MR, Tosteson TD, Morris JS, Demidenko E, Mott LA, Heaney J (2004). Incidence of transitional cell carcinoma of the bladder and arsenic exposure in New Hampshire.. Cancer Causes Control.

[r14] Kumar A, Adak P, Gurian PL, Lockwood JR (2010). Arsenic exposure in U.S. public and domestic drinking water supplies: a comparative risk assessment.. J Expo Sci Environ Epidemiol.

[r15] Kurttio P, Pukkala E, Kahelin H, Auvinen A, Pekkanen J. (1999). Arsenic concentrations in well water and risk of bladder and kidney cancer in Finland.. Environ Health Perspect.

[r16] Luben T, Nuckols JR, Lynberg M, Mendola P, Wolf J (2004). Feasibility of matching study participant residence with a specific water utility in epidemiologic studies investigating exposure to disinfection byproducts.. Epidemiology.

[r17] Luben TJ, Nuckols JR, Mosley BS, Hobbs C, Reif JS (2008). Maternal exposure to water disinfection by-products during gestation and risk of hypospadias.. Occup Environ Med.

[r18] Lubin JH, Colt JS, Camann D, Davis S, Cerhan J, Severson RK (2004). Epidemiologic evaluation of measurement data in the presence of detection limits.. Environ Health Perspect.

[r19] Marshall G, Ferreccio C, Yuan Y, Bates MN, Steinmaus C, Selvin S (2007). Fifty-year study of lung and bladder cancer mortality in Chile related to arsenic in drinking water.. J Natl Cancer Inst.

[r20] Meliker JR, Slotnick MJ, Avruskin GA, Kaufmann A, Fedewa SA, Goovaerts P (2007). Individual lifetime exposure to inorganic arsenic using a space–time information system.. Int Arch Occup Environ Health.

[r21] Mink PJ, Alexander DD, Barraj LM, Kelsh MA, Tsuji JS (2008). Low-level arsenic exposure in drinking water and bladder cancer: a review and meta-analysis.. Regul Toxicol Pharmacol.

[r22] Nuckols JR, Langlois P, Lynberg M, Luben T (2004a). Linking Geographic Water Utility Data with Study Participant Residences from the National Birth Defect Prevention Study.

[r23] Nuckols JR, Ward MH, Jarup L (2004b). Using geographic information systems for exposure assessment in environmental epidemiology studies.. Environ Health Perspect.

[r24] Steinmaus C, Yuan Y, Bates MN, Smith AH (2003). Case–control study of bladder cancer and drinking water arsenic in the western United States.. Am J Epidemiol.

[r25] Stewart W, Correa-Villasenor A. (1991). False positive exposure errors and low exposure prevalence in community-based case–control studies.. Appl Occup Environ Hyg.

[r26] Straif K, Benbrahim-Tallaa L, Baan R, Grosse Y, Secretan B, El Ghissassi F (2009). A review of human carcinogens. Part C: metals, arsenic, dusts, and fibres.. Lancet Oncol.

[r27] Subcommittee on Arsenic in Drinking Water, Committee on Toxicology, Board on Environmental Studies and Toxicology, Commission on Life Sciences, National Research Council (1999). Arsenic in Drinking Water.

[r28] Subcommittee to Update the 1999 Arsenic in Drinking Water Report, Committee on Toxicology, Board on Environmental Studies and Toxicology, Division on Earth and Life Sciences, National Research Council (2001). Arsenic in Drinking Water—2001 Update.

[r29] USCB (U.S. Census Bureau) (2000). Cartographic boundary files.. http://www.census.gov/geo/www/cob/bdy_files.html.

[r30] U.S. EPA (U.S. Environmental Protection Agency) (2000). Arsenic Occurrence in Public Drinking Water Supplies. EPA 815-R-00-023.

[r31] U.S. EPA (U.S. Environmental Protection Agency) (2008). SDWIS (Safe Drinking Water Information System).. http://www.epa.gov/enviro/html/sdwis/sdwis_ov.html.

[r32] U.S. EPA (U.S. Environmental Protection Agency) (2009). Underground Storage Tanks. Map of EPA Regions.. http://www.epa.gov/OUST/regions/regmap.htm.

[r33] USGS (U.S. Geological Survey) (2002). Map Layer Info. Aquifers of Alluvial and Glacial Origin—U.S. Geological Survey.. http://nationalatlas.gov/mld/alvaqfp.html.

[r34] USGS (U.S. Geological Survey) (2008). Map Layer Info. Principal Aquifers of the 48 Conterminous United States, Hawaii, Puerto Rico, and the U.S. Virgin Islands.. http://nationalatlas.gov/mld/aquifrp.html.

[r35] Watermolen J (2005). U.S. Geological Survey, 200506, 1:2,000,000-Scale Hydrologic Unit Boundaries.

