# Supplementary material for: Estimating Water Supply Arsenic Levels in the New England Bladder Cancer Study
Source: Environ Health Perspect. 2011 Mar 21;119(9):1279–85. doi: 10.1289/ehp.1002345 (PMC3230387; doi:10.1289/ehp.1002345)
Supplement: (444 KB) PDF [file ehp.1002345.s001.pdf]

SUPPLEMENTAL MATERIALS

Estimating Water Supply Arsenic Levels in the New England Bladder Cancer Study

John R. Nuckols, Laura E. Beane Freeman, Jay H. Lubin, Matthew S. Airola, Dalsu Baris, Joseph D. Ayotte, Anne Taylor, Chris Paulu, Margaret R. Karagas, Joanne Colt, Mary H. Ward, An-Tsun Huang, William Bress, Sai Cherala, Debra T. Silverman, Kenneth P. Cantor

TABLE OF CONTENTS

TITLE	MANUSCRIPT PAGE
SM_Figure 1. Location of case and control ascertainment (primary study area)	8
SM_Figure 2. Location of participant residence at time of enrollment (current home) by water supply type.	8
SM_Figure 3. Location of residential history (past homes) by assigned water supply type.	8
SM_Table 1. Historical public water supply measurement data available by time period and state.	10
SM_Table 2. Detailed description of prediction models used to assign arsenic concentration in the water supply of study participant residences and workplaces.	11
SM_Table 3. Variables used in the predictive models for arsenic concentration in wells using the bedrock and unconsolidated materials aquifers of New England.	13

Corresponding author: Professor J.R. Nuckols, PhD, Department of Environmental and Radiological Health Sciences, Colorado State University, c/o10916 Wickshire Way, Rockville, MD 20852; Phone number: (970) 218-4757; FAX number (301) 560-8589; email: jnuckols@colostate.edu

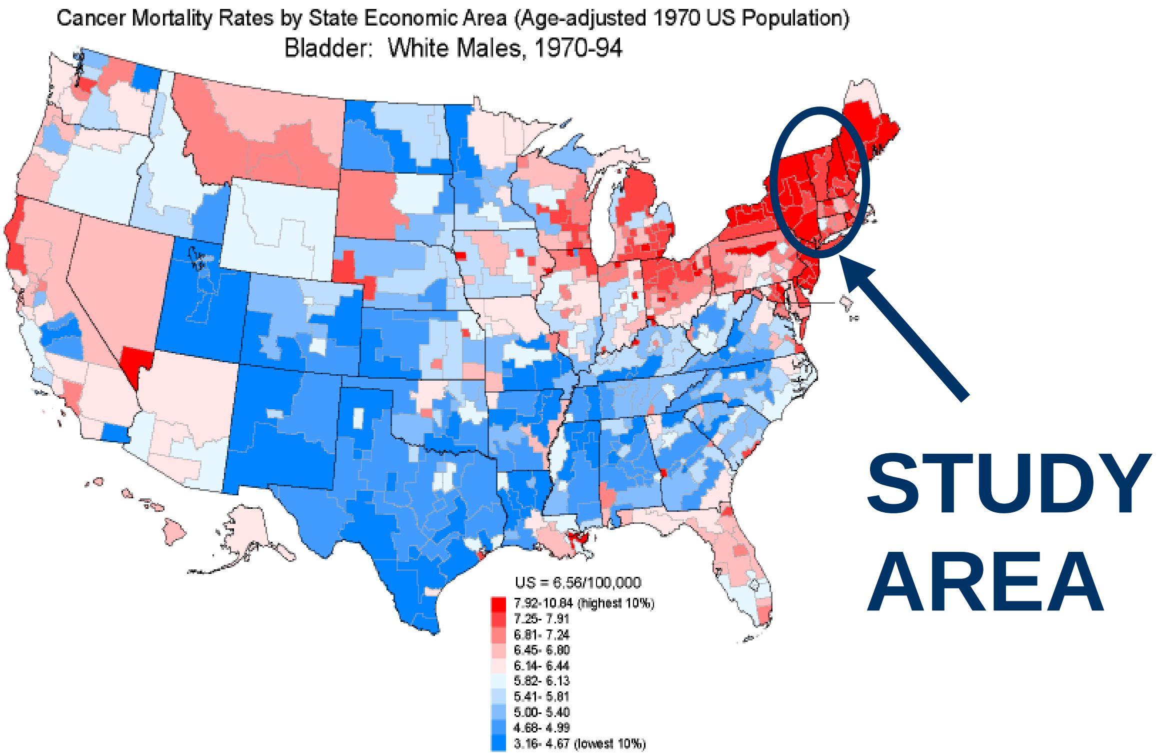

SM-Figure 1. Location of case and control ascertainment (primary study area), New England Bladder Cancer Study.

Source of base map: Atlas of Cancer Mortality in the United States, 1950-1994, NCI (http://www3.cancer.gov/atlasplus/new.html)

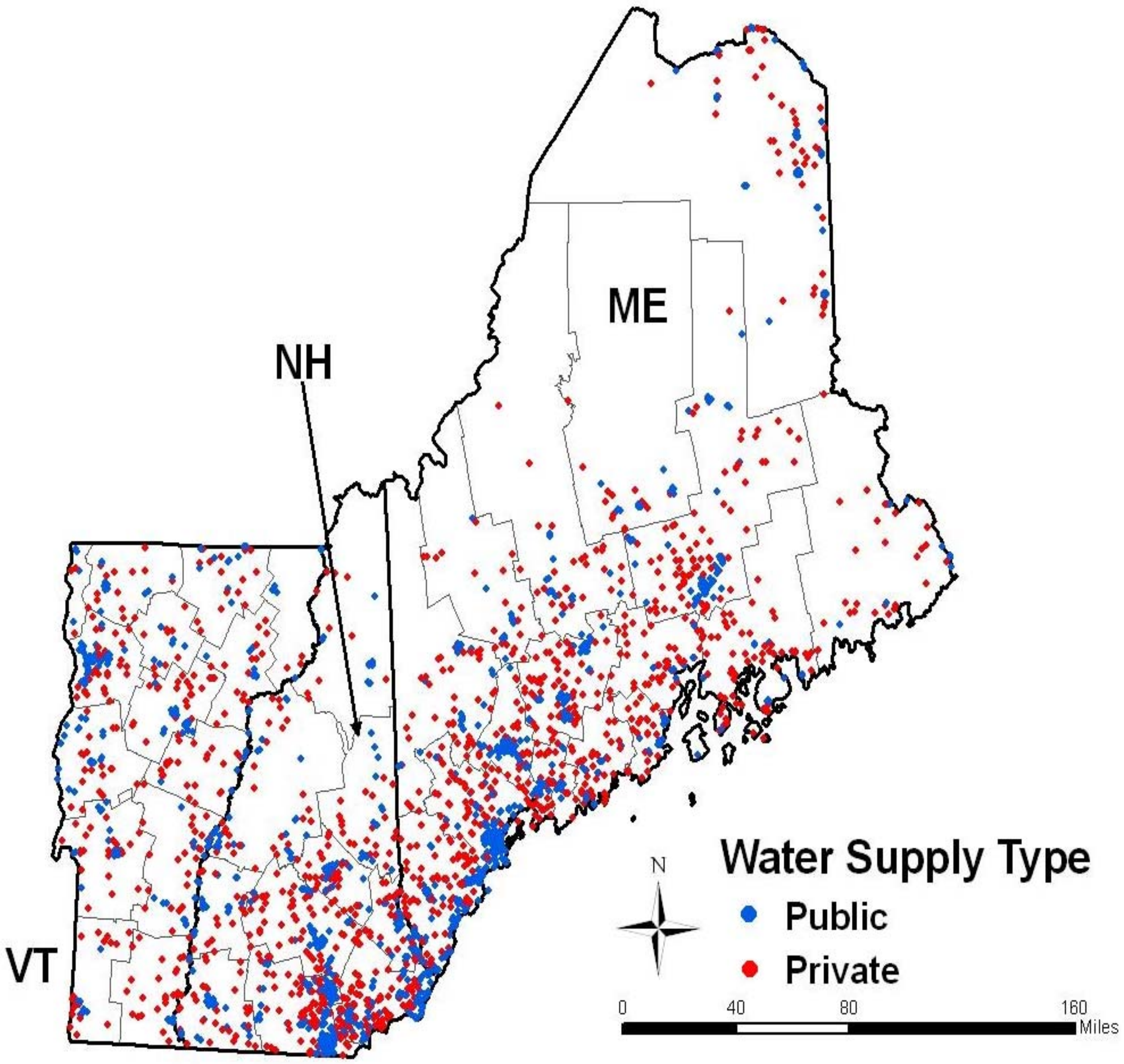

SM-Figure 2. Location of participant residence at time of enrollment (current home) by water supply type, New England Bladder Cancer Study.

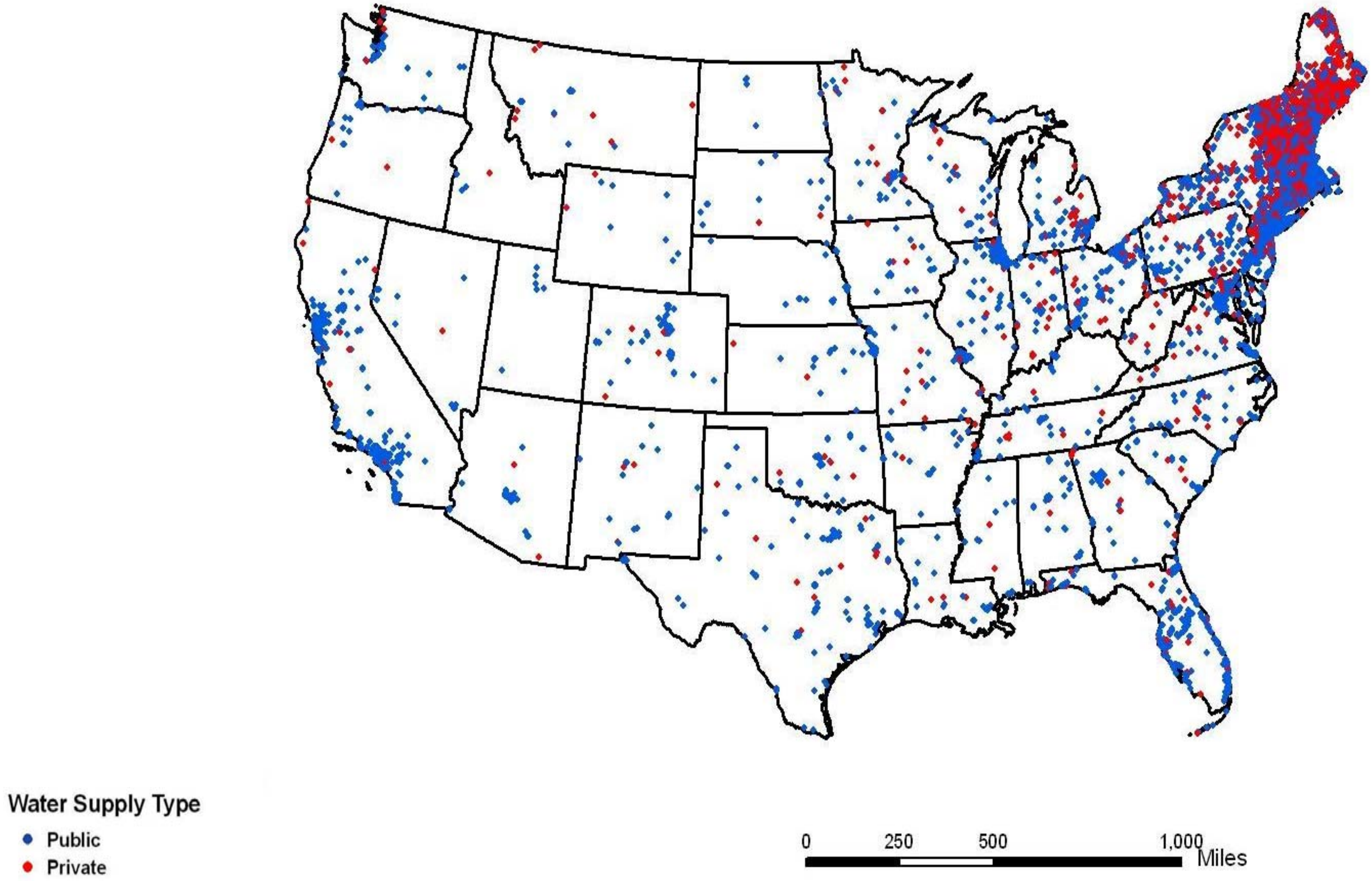

SM-Figure 3. Location of residential history (past homes) by assigned water supply type, study participants in the New England Bladder Cancer Study.

SM-Table 1. Historical public water supply measurement data available by time period and state, New England Health and Environment Study.

Historical Public Utility Arsenic Measurements by State[1]				
State	Total Observations[2]	Earliest	Latest	Number of Utilities with at least one observation
AK	676	1991	1997	270
AL	1787	1984	2000	323
AR	634	1996	1998	400
AZ	2561	1988	1998	536
CA	5531	1980	2000	662
CT	1626	2003	2008	19
FL	1639	2004	2006	1281
IL	2724	1993	2000	914
IN	696	1996	1999	466
KS	1912	1991	1997	516
MA	375	1990	2005	27
ME	1317	1975	2005	145
MI	290	1993	1997	258
MN	1277	1993	1997	652
MO	36	1995	1997	30
MT	1434	1980	1992	324
NC	7246	1980	2000	1102
ND	179	1993	1996	177
NH	522	1984	2005	104
NJ	788	1993	1997	332
NM	2652	1980	2000	417
NV	148	1991	1997	147
NY	548	1997	2008	106
OH	3930	1980	1994	767
OK	588	1986	1998	448
OR	1512	1990	1998	472
PA	3685	2003	2008	1285
TX	5911	1994	1999	2767
UT	2499	1980	1999	277
VT	194	1973	2001	107
**Total**	**54917**	**1973**	**2008**	**15331**

[1] Measurements in MA, ME, NH and VT exclude current home measurements made during the study
[2] Includes observations below a detection limit.

SM-Table 2. Descriptions of prediction models developed for assigning arsenic to residences and workplaces, New England Health and Environment Study.

**Model Categories**	**Description of Measurements**[1]	**Model Description**
**Public Water Supply (4-State Region)**[2]		
**Utilities with Measurements Above Detection**		
Surface Water Source	N=1,254 (656 BDL; 598 ADL)[3], K= 59	Model: $\ln(As) = \sum_u \beta_u x_u + \varepsilon$ where x_u is a zero/one indicator variable for u=1, …, K utilities, β_u is the parameter estimate, and ε is the error, assumed to be normally distributed with mean 0 and variance σ^2.
Ground Water Source - Unconsolidated Aquifer[4]	N= 497 (236 BDL; 261 ADL), K= 46	
Ground Water Source – Unspecified Aquifer	N = 838 (384 BDL; 444 ADL), K=109	
Mixed – Surface and Groundwater Source (Unspecified)	N = 599 (211 BDL; 388 ADL), K=27	
**Utilities with No Measurements or No Measurements Above Detection**		
Utilities where all measurement data was below laboratory limit of detection, regardless of water source type	N =339, K=44	For each utility, K, we assumed the number of measurements observed below the detection limit, given n total measurements, was binomially distributed and derived a value for b_{uk} in the above model.
Utilities with no measurements, by water source type[5]	S = 4, N=3482 (1775 BDL; 1707 ADL)	Model: $\ln(As) = \sum_s \beta_s x_s + \varepsilon$ State-specific (s = 1,…, S) model. x_s is a zero/one indicator variable for State s and β_s is the parameter estimate (service-population-weighted mean measurement data for s), and ε is the error, assumed to be normally distributed, with mean 0 and variance σ^2.
**Public Water Supply (outside 4-State Region)**[6]		
**Utilities with Measurements Above Detection**		
Ground water	N = 17,717 (7,717 BDL; 10,000 ADL); K= 4,832	Model: $\ln(As) = \sum_u \beta_u x_u + \varepsilon$ Same as utility-specific model within 4-State Region described above
Surface water	N = 6,385 (4,507 BDL; 1,878 ADL); K= 703	
**Utilities with No Measurements or No Measurements Above Detection**		
Utilities where all measurement data was below laboratory limit of detection, regardless of water source type	N = 993; K = 187	For each utility, K, we assumed the number of measurements observed below the detection limit, given n total measurements, was binomially distributed and derived a value for b_{uk} in the above model.
Utilities with no measurements, by water source type (surface or ground)	S = 26 , N =52,509 (40,631 BDL; 11,878 ADL)	Model: $\ln(As) = \sum_s \beta_s x_s + \varepsilon$ Same as state-specific model within 4-State Region described above. If insufficient statewide data, model was applied using data from USEPA Region where the utility is located.

Continuation from Supple. Materials, P.6. SM-Table 2. Descriptions of prediction models developed for assigning arsenic to residences and workplaces, New England Health and Environment Study.

Model Categories	Description of Measurements	Model Description
**Private Water Supply (6-State Region)** [7]		
Wells - Bedrock Aquifer Source	N = 3,527	Model: $\ln(As) = \beta x + \varepsilon$<br>Model includes 12 geographic-based variables (x) based on geologic provinces, litho chemistry and surficial geology of bedrock units (Table 2).
Wells - Unconsolidated Materials Aquifer Source	N = 1,557	Model: $\ln(As) = \beta x + \varepsilon$<br>Model includes 13 geographic-based variables (x) based on geologic provinces, litho chemistry and surficial geology of bedrock units (Table 2).
**Private Water Supply (outside 6-State Region)**		
USGS Hydroregion Subbasin (Watermolen, 2005) modeling unit	N =18,651; H = 934 subbasins where residences/workplaces located	Model: $\ln(As) = \sum_h \beta_h x_h + \varepsilon$<br>h=1,...,H, where x_h zero/one indicator variables for hydroregion h, β_h is the parameter estimate (mean measurement data for h), and ε is the error, derived from normally distributed measurements N, mean 0 and variance σ^2
Principal Aquifer Modeling Unit	N =15,687, P = 64 Principal Aquifer boundaries (USGS 2008) where hydroregion subbasins with residences/workplaces located	Model: $\ln(As) = \sum_p \beta_p x_p + \varepsilon$<br>USGS Principal Aquifer-specific model using measurements from all study hydroregion subbasin wells located within each aquifer boundary

[1] N – number of samples; K - number of utilities; S – number of states in USA

[2] Study states of Maine, New Hampshire, and Vermont plus Massachusetts.

[3] BDL=Below Detection Limit; ADL=Above Detection Limit. If the detection limit was not reported in the measurement (e.g., listed only as BDL) then the following assignments for the detection limit were made: Prior to 1995, the limit of detection was assigned to be 5 µg/L; from 1995-2000, the detection limit was assigned to be 1µg/L and from 2001 forward, they were assigned to be 0.5 µg/L based on reported detection limits from other utilities.

[4] Excludes one utility with unconsolidated source type, which had an unusually large number of measurements, n=462, of which 185 were BDL and 277 were ADL. A separate parameter estimate was developed for this utility.

[5] Each of the 4 states had the potential to have each type of model (surface, unconsolidated, overall ground, mixed).

[6] Only current information for source type was known. Therefore, the current source type was therefore applied to all time periods.

[7] Study states Maine, New Hampshire, and Vermont + Massachusetts, Connecticut, and Rhode Island. Modeling area used in predictive model for bedrock aquifer in Ayotte et al, 2006

SM-Table 3. Variables used in the predictive models for arsenic concentration in wells using the bedrock and unconsolidated materials aquifers of New England, New England Health and Environment Study.

Arsenic sources -geologic provinces	Variable Type	Bedrock Model	Unconsol. Model
Avalon Belt	binary polygon	X	X
Bronson Hill Belt	binary polygon	X	
Eugeosyncline Sequence	binary polygon	X	
Mesozoic Basin	binary polygon	X	X
Waits River Basin	binary polygon	X	
Grenville – Grenville Belt geologic province	binary polygon	X	X
NarrBasin – Narragansett Basin geologic province	binary polygon		X
**Arsenic sources – lithochemistry**			
pelitic rocks (Bronson Hill)	binary polygon	X	
peraluminous granite (New Hampshire Maine Sequence)	binary polygon	X	
mafic rocks (Narragansett Basin)	binary polygon	X	
MaficCM – Mafic igneous rocks of the Coastal Maine province	binary polygon	X	
PeliticCM – Pelitic rocks of the Coastal Maine province	binary polygon	X	
MaficNHME – Mafic igneous rocks of the New Hampshire – Maine Sequence province	binary polygon		X
**Arsenic sources -bedrock geologic units**			
Concord Granite (Dc1m, granite)	binary polygon	X	
Madrid Fm. (DSm), metamorphic	binary polygon	X	X
Rindgemere Fm., lower member (DSrb, metamorphic)	binary polygon	X	
Berwick Fm., calcareous member (SObc), Calcpelite	binary polygon	X	
Eliot Fm., Calef member (SOec), metamorphic	binary polygon	X	
Kittery Fm., (SOk), metamorphic	binary polygon	X	
Perry Mountain Fm. (Sp, metamorphic)	binary polygon	X	
Rangeley Fm., lower part, (Srl), metamorphic	binary polygon	X	X
Sangerville Fm., (Sspm, metamorphic)	binary polygon	X	
Waterville Fm., (Sw, metamorphic)	binary polygon	X	
Kittery Fm., (SZk, metamorphic)	binary polygon	X	
Massabesic Gneiss Complex (Zmz, granite)	binary polygon	X	
Ayer Granodiorite (Sa2x, Granite)	binary polygon	X	
Fitchburg Complex (Dfgds, Granite)	binary polygon	X	
Spaulding Tonalite (Ds16, Granite)	binary polygon	X	
Perry Mountain Formation (Sp, Metamorphic)	binary polygon	X	
Eliot Formation (Soe, Metamorphic)	binary polygon		X
Oakdale Formation (SO, Calcpelite)	binary polygon		X
Berwich Formation (Sob, Metamorphic)	binary polygon	X	
**Arsenic sources -integrated natural and anthropogenic**			
stream sediment arsenic, (ln) mg kg-1	Continuous grid	X	X
**Geochemistry**			
**Pleistocene marine inundation**	binary polygon	X	X
intrusive granitic pluton category (within 3 km of pluton)	binary polygon	X	
hydrologic processes and land use			
developed land flag (cut point of 33% within 1.0 Km)	binary grid	X	X
elevation (1:24,000 scale DEM, m)	continuous grid	X	X
population density (persons km-2)	continuous grid	X	X
precipitation, mm yr-1	continuous grid	X	
water bodies (% area in 1000 m radius buffer)	continuous grid	X	X
**Geographic**			
State	Categorical		X
New England Township	Categorical	X	X
Proximity index to arsenic measurements greater than 5.0 µg/L (kilometers)	Categorical		X
Estimate of bedrock model at same location	Continuous		X
